# Somatic Genomic Variations in Early Human Prenatal Development

**DOI:** 10.2174/138920210793175967

**Published:** 2010-09

**Authors:** Caroline Robberecht, Evelyne Vanneste, Anne Pexsters, Thomas D’Hooghe, Thierry Voet, Joris R. Vermeesch

**Affiliations:** 1 Center for Human Genetics, Leuven, Belgium; 2 University Hospitals Leuven, Leuven University Fertility Center, Leuven, Belgium

**Keywords:** Aneuploidy, chromosome aberrations, embryonic and fetal development, somatic genomic variations.

## Abstract

Only 25 to 30% of conceptions result in a live birth. There is mounting evidence that the cause for this low fecundity is an extremely high incidence of chromosomal rearrangements occurring in the cleavage stage embryo. In this review, we gather all recent evidence for an extraordinary degree of mosaicisms in early embryogenesis. The presence of the rearrangements seen in the cleavage stage embryos can explain the origins of the placental mosaicisms seen during chorion villi sampling as well as the chromosomal anomalies seen in early miscarriages. Whereas these rearrangements often lead to implantation failure and early miscarriages, natural selection of the fittest cells in the embryo is the likely mechanism leading to healthy fetuses.

## INTRODUCTION

Only 25 to 30% of conceptions result in a live birth [1]. The other 70-75% arrest at different stages of pregnancy, largely due to chromosome aberrations. Some aberrations are commonly found in prenatal diagnosis and analyses of miscarriages, but many are never detected or are only detected in a small percentage of the fetal cells. This shows that these rare aberrations do indeed occur at early stages of development, but are selected against and are likely embryonic lethal.

## CHROMOSOME ERRORS IN HUMAN EMBRYOS

Chromosome instability is common in human *in vitro* fertilized (IVF) cleavage stage embryos. Over the past two decades due to the application of even better techniques for the analysis of the chromosome content of single blastomeres chromosome instability has come to light.

Until recently, several studies of normally developing, good quality preimplantation embryos with fluorescent *in situ* hybridization (FISH) have shown that 45-70% of these embryos have a chromosomal abnormality (aneuploidy and/or polyploidy) in at least one cell [2-5]. When the results of different blastomeres of the same embryo are combined 25% to more than 60% of embryos show mosaicism [2, 6-9]. These numbers are bound by a few limiting factors. FISH depends on fluorochromes that can be used on a nucleus simultaneously, usually 4 or 5. After analysis of the signals and washing off the probes, a second round with different probes can bring the number of chromosomes tested up to about 7 to 9. The most frequent protocols used only 3 to 6 probes, targeting the chromosomes most commonly found in spontaneous abortions. All other chromosomes are inherently missed and would raise the aberration rate if included. Detection of segmental aberrations is also not possible with this standard FISH setup, because the probes used on embryos bind to alpha-satellite sequences at the centromere or at a nearby locus. Detection of a third signal is generally interpreted as a trisomy for that chromosome, even if the cause could infact be a translocation, a marker, or another aberration.

Since the introduction of comparative genomic hybridization (CGH), it has been possible to determine the copy number of all chromosomes in the nucleus of a blastomere [10, 11]. Because a measurement of the fluorescence is made over the entire length of the chromosome, it becomes possible to detect aneuploidy of segments of chromosomes. With this technique, the limit of detection per single cell lies around 40Mb, due to the amplification of DNA necessary to obtain a sufficient amount of DNA [12]. In 2000, two groups studied 12 normally developing embryos with CGH [13, 14] each. This amounted to ~65 available blastomeres per study. Both groups found abnormalities in 75% of embryos (9/12), confirming the high chromosome aberration rate from previous FISH studies. Most of these abnormal embryos (7/9 & 8/9) had aneuploidy in some but not all analyzed cells, showing a high frequency of mosaicism (78% & 89%). It was also demonstrated that the chromosomes not probed by FISH are indeed involved in cleavage stage aberrations. With the use of CGH, segmental aberrations were detected in early human embryos for the first time. Some clearly originated from post-zygotic events, with deletions and complementary duplications or even amplifications in different cells from the same embryo. Later studies confirmed both the high aneuploidy rate (~60%) and the segmental aberrations in cleavage stage embryos [15, 16].

Array CGH uses the same principle as metaphase CGH, with fluorescent labeling of the complete genomic DNA of patient and reference and measurement of patient/reference ratios. The technique was tested on the genomic content of single fibroblasts, EBVs (Ebstein Barr virus-transformed lymphoblastoid cells) and blastomeres. In a study of 23 normally developing, good quality preimplantation embryos (165 blastomeres), 91% (21/23) had chromosomal abnormalities. All abnormal embryos were mosaic, with combinations of diploid and aneuploid cells (~50% of the embryos) or different and multiple types of aneuploidies. As with CGH, segmental aberrations could be detected, but they were unexpectedly found in 70% of the embryos. When array CGH was used in a study to screen 41 embryos from patients with at least 7 recurrent IVF failures by assessing 2 blastomeres per embryo, abnormalities were detected in 60% of embryos [17]. FISH on additional blastomeres from affected embryos showed that the aberrations could not be confirmed in some instances in all blastomeres, pointing to mosaicism of these aberrations. A lower percentage of segmental aberrations was observed (5%). The presence of these segmental aneuploidies could be extrapolated from certain rare findings from prenatal diagnosis, spontaneous abortions or affected patients [18-20], but had not been proven in early human embryos before.

With such a high percentage of abnormalities found, the question arises whether these are true findings that would be seen in all early conceptions, or the result of a specific study group. Many studies on cleavage stage embryos used patients referred for fertility problems, but the same aberration rates were shown in the analysis of embryos from fertile patients undergoing PGD (Preimplantation Genetic Diagnosis) for sex selection, a single gene disorder or a familial microdeletion syndrome, without the indication of infertility [3, 21]. Embryos from patients with a high maternal age could have more aberrations because of a high meiotic error rate. However, the finding that many of the abnormal embryos are a mixture of normal and aneuploid cells points towards a mitotic cause for the majority of those chromosomal errors.

## GENOMIC VARIATIONS IN PRENATAL DEVELOPMENT

**Fig. (1):**
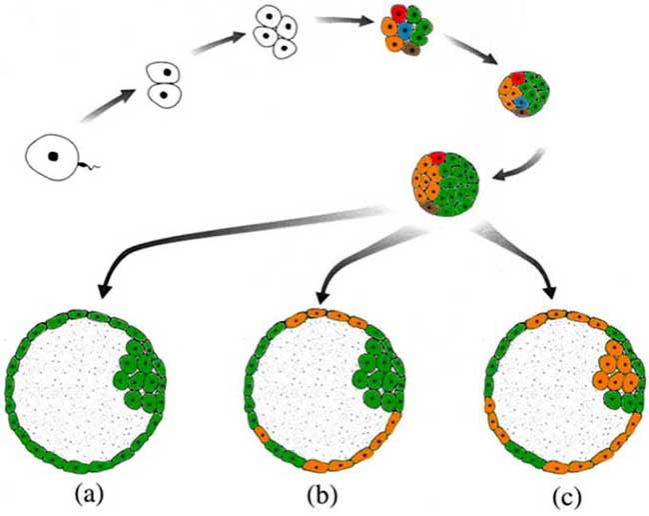
The development of a human embryo to the blastocyst stage. The different colors at the eight cell stage represent mosaicism of normal blastomeres (green) and blastomeres carrying mitotically derived aneuploidies and mitotic structural aberrations (orange, red, blue and brown). When the embryo reaches blastocyst stage, the aberrant cells can be lost due to negative selection (**a**); they can segregate to the trophectoderm only, leading to confined placental mosaicism (**b**); or they can be found in both the inner cell mass and the trophectoderm resulting in an embryo that is affected in certain tissues (**c**).

Many of the chromosomal aberrations seen in cleavage stage embryos are rarely, if ever, observed at the clinical stage of pregnancy. These include most monosomies, certain trisomies and the majority of segmental aberrations. Embryos with these aberrations or with a multitude of aberrations in different cells, as seen in the studies of Voullaire *et al*. (2000) [13], Wells & Delhanty (2000) [14] and Vanneste *et al*. (2009) [21], likely arrest before implantation. Aside from selection against abnormal embryos, it is also plausible that there is active selection against individual anomalous blastomeres or the possibility of active recruitment of these abnormal blastomeres towards the placental tissues (Fig. **1**). Several studies have shown that the degree of mosaicism and the aneuploidy rate are lower at blastocyst stage and that an embryo diagnosed as aneuploid at day 3 by FISH may still lead to a normally developing euploid blastocyst [22-25]. However, a comparison of cells from the inner cell mass and the trophectoderm indicates there is no evidence in blastocysts for preferential segregation of aberrant blastomeres to the trophectoderm.

The natural selection against abnormal embryos during the first days and weeks of gestation is reflected in a lower percentage of chromosomal abnormalities found in spontaneous abortions (50-60%) [26-28] compared to cleavage stage embryos (60-90%). The chromosomal constitution of spontaneous abortion material has been studied for decades and all trisomies have been observed at least once. A number of trisomies are more commonly found (tris. 16, 21, 22), indicating that these are less detrimental during embryonic development. Autosomal monosomies are rarely detected at the clinical stage of pregnancy [27, 29-31]. The fact that these monosomy cases were often mosaic shows that these embryos survived through the first trimester because the monosomy was confined to the placental tissues or was only present in a smaller number of embryonic cells. Segmental aberrations appear in 4-7% of spontaneous abortions [31-35], which does not correlate with the high percentage found in cleavage stage embryos. The striking difference cannot be attributed to a difference in resolutions for array CGH and G-banding, as the detection limit lies around 10 Mb in array CGH on single cell material and around 5 Mb for G-banding on genomic miscarriage DNA. Several studies using array CGH [36-38] on products of conception (POC) have shown that small deletions and duplications are not a frequent cause of miscarriage and do not significantly increase the percentage of segmental aberrations in spontaneous abortions. Many of the analyzed cleavage stage embryos with segmental aberrations had multiple and/or complementary aberrations affecting multiple cells and in combination with the other aneuploidies found in these embryos they likely arrested before implantation. 

The presence of mosaicism has not been extensively investigated in spontaneous abortion samples. A study by Vorsanova *et al*. (2005) [39] using interphase FISH with probes for half of all chromosomes showed 30% mosaicism. This number is in contrast with mosaicism rates from other recent studies: 6% by Martinez *et al*. (2010) [40], and 3% by Robberecht *et al*. (2009) [37]. It is also much higher than the 2-5% as cited in the past [27, 41]. The lower percentages might be due to the exclusion of cases with mosaicism of aberrant female cells and normal female cells, as these cases are thought to be the result of maternal contamination. Another cause could be the selective nature of biopsies taken from products of conception. This would mean that tissue-specific or very low percentage aneuploidy might be missed. It warrants further research to confirm these results.

Prenatal diagnosis by chorionic villus sampling (CVS) or amniocentesis provides information on the chromosomal aberrations found beyond the first trimester. The percentage of chromosomal abnormalities detected by CVS lies around 5% [42, 43]. However, this number is influenced by the specific indications for which CVS is performed: maternal age, presence of familial chromosome rearrangements or abnormalities seen on ultrasound. The incidence of mosaicism for chromosomal aberrations in CVS has been reported to be 0.8-2% [42, 44, 45]. Only about 10-25% of these mosaics are confirmed in amniotic fluid (AF) or fetal blood [42, 46, 47]. They constitute cases of generalized mosaicism with true fetal involvement. The remaining 90% are categorized as confined placental mosaicism (CPM). The effect of CPM on the pregnancy varies greatly: some fetuses develop without problems; while others suffer from intra uterine growth retardation or spontaneously abort. One must however be cautious with CPM, because a number of cases without detection of the aberration in AF were shown to contain cryptic fetal mosaicism. Here, the trisomy was present in one or more tissues other than those sampled by AF cells, which did not always lead to a clinical phenotype [46, 48-50]. The origin of mosaicism at prenatal diagnosis has been investigated and both meiotic and postzygotic mitotic mechanisms were observed [46, 51, 52]. For cases of meiosis I or II origin, a trisomy rescue event resulted in the diploid cell line. In theory, this should lead to uniparental disomy in one third of the cases. For certain chromosomes (e. g. 11, 15) this causes known genomic imprinting related defects. For other chromosomes, for instance chromosome 16, the effect of the UPD remains unclear, as any phenotypic abnormalities seen in the fetus could be caused by placental insufficiency; these abnormalities are the same as those seen in fetuses with biparental chromosome 16 inheritance and UPD16 has been found in fetuses that developed normally to term [53]. UPD may also lead to a loss of heterozygosity that could in turn activate recessive disease causing alleles. The underlying mechanism of origin and the frequency with which a CPM appears, differ from chromosome to chromosome and depend on the viability of the specific chromosome and the percentage and different kinds of cell lineages that are affected [46, 51, 52].

## CONCLUSIONS

Studies using FISH, CGH and array CGH have clearly demonstrated that up to 90% of IVF embryos start with a burden of mitotic errors from the first cleavage divisions, including whole chromosomes and segmental aneuploidies. These embryos arrest or fail to implant and make up the first 30% of embryos lost after conception [1]. There is hardly any data about the fate of embryos during the early weeks of pregnancy, after implantation but before clinical detection or even before the first missed menstrual period. Several studies of hysterectomy specimens and studies using daily urine hCG measurements, each in the normal fertile population, show that a further 30% of embryos are lost at this stage [1]. However, the genetic constitution of these embryos has not been investigated. One way to achieve this would be to do detection and follow-up of biochemical pregnancies by hCG level counts until a stagnation or drop in these levels indicates an early miscarriage. A hysteroscopic embryoscopy can then be used to evaluate the morphology of the embryo *in utero* and to take a sample of embryonic tissue. This would allow both the correlation of morphologic abnormalities with specific genetic conditions and the determination of the type of aberrations, both large and small, in embryos at this early stage. Taking an accurate biopsy of such early implantation embryos will be technically challenging and it will be difficult to avoid maternal contamination. Another approach may be the *in vitro* culture of blastocysts. The growth and interaction of blastocysts with stromal cells in relation to their chromosomal constitution could then be followed [54, 55].

With a chromosome abnormality rate of 50-60% in spontaneous abortion samples and the detection of anatomic, hormonal or immunological causes in some recurrent abortion cases, 40% of miscarriages still remain unexplained. Some could be the result of single gene disorders, both caused by mutations or chromosomal aberrations. These genomic errors occur *de novo* by chance and will likely be rare findings. One could also speculate that some of the copy number variations (CNV) commonly found in the general population are not tolerable in certain combinations. A Hardy-Weinberg disequilibrium could for example point to the lethal effect of a homozygous deletion or amplification of a specific CNV [56-58]. Future research is required to further elucidate the causes and mechanisms behind the many aberrations encountered throughout embryonic and fetal development [59-61].
